# Effect of transportation distances, seasons and crate microclimate on broiler chicken production losses

**DOI:** 10.1371/journal.pone.0232004

**Published:** 2020-04-22

**Authors:** Vinícius M. dos Santos, Bruno S. L. Dallago, Aline M. C. Racanicci, Ângela P. Santana, Roger I. Cue, Francisco E. M. Bernal

**Affiliations:** 1 Federal Institute of Brasília, Planaltina, Brasília/DF, Brazil; 2 Faculty of Agronomy and Veterinary Medicine, University of Brasília, Asa Norte, Brasília/DF, Brazil; 3 Department of Animal Science, McGill University, Canada; Tokat Gaziosmanpasa University, TURKEY

## Abstract

The goal of this research was to evaluate the microclimate (temperature, relative humidity and ECI–enthalpy comfort index) of commercial loads of broiler chickens at different transport distances: Dist15 (15 km on average) and Dist90 (90 km on average) in the summer and winter seasons and their effects on the production parameters body weight difference (BWD), mortality (%) and bruising prevalence (%). Twelve broiler loads were monitored using dataloggers to record temperature and humidity, with a total of 24 target crates per load. The experiment followed a factorial design [2 seasons (rainy and dry) × 2 distances (Dist15 and Dist90)] with a randomized complete block arrangement, 3 sexes (all males, all females, or mixed shipments) and one shipment per combination. BWD had a heterogeneous distribution throughout the load, and this distribution was not significantly correlated with the mean ECI measured during transport at 12 positions along the load. In terms of comfort, summer is the most critical period for broiler transport. In the interaction between rainy season and Dist90, the highest ECI was scored in the lethal zone (where physiological mechanisms are not enough to control body temperature). Mortality during the rainy season was not significantly different between distances. However, during the dry season, mortality was twice as high as broilers that travelled for 15 km. The prevalence of bruising on carcasses was not affected by the interaction between season and distance. As we know, broiler chicken performance, during transport, can be also related to road conditions, being hard to evaluate the real impact of seasons and distances on animal welfare. Load microclimate can compromise broiler chicken welfare during transport and it does not necessary reflect significant losses pre and post-slaughter.

## Introduction

The transport of broilers is considered a critical point in the production chain [1] given the possible implications for broiler welfare [2]. During pretransport handling, broilers are exposed to stressful conditions that can persist and even intensify throughout transport from the farm to the slaughterhouse. Long-term water and feed deprivation have been correlated with yield losses at slaughter [3–5], and factors related to the vehicle, such as vibration, impact and road noise, also represent considerable sources of stress [6] with consequent losses of yield parameters. In addition, variations in climatic conditions during transport, such as changes in temperature, relative humidity and air flow inside the cargo bay, are important stressors for broilers [7] and are not fully controllable in the vehicles current used to broiler transportation.

Climatic conditions during transport can influence the microclimate of shipments. High environmental temperature and high humidity promote changes in broiler behavior to assume a better body position for improved heat transfer. The chicken stretches its wings, opens its mouth, lowers it head and tries to touch its breast to the floor. In addition, it changes its body metabolism to increase respiratory rates in an attempt to reduce the adverse effects of heat stress [8,9]. This physiological response (increased respiratory rates) increases the temperature and humidity in the microclimate and makes it even more difficult to lose body heat through panting. High temperature and high humidity can be sufficiently dangerous to cause pH disequilibrium with respiratory alkalosis due to marked CO_2_ elimination and can ultimately cause death [10]. Inevitably, the heat produced by broiler metabolism is retained (at least partially) in the load, and its displacement dynamics depend directly on the speed and intensity of the air flow inside the cargo bay [11].

In Brazil, due to climatic attributes, broiler chickens are usually subjected to thermal variations that lead to heat stress during transport [12]. However, some regions of the country and/or certain periods of the year, typically winter, present conditions of low temperature and low humidity. In these conditions, to maintain warmth, the broilers become less active in the crate in an attempt to transfer heat by conduction, and depending on the duration of exposure to these conditions, muscle glycogen catabolism may increase, resulting in changes in meat quality [13].

The consequences of microclimatic factors are almost always described as a function of broiler performance at slaughter, such as carcass yield (quantity of meat) [14,15] and carcass quality (e.g., presence of bruises) [16]. However, with a focus on animal welfare, these analyses must also consider and evaluate the degree of compromise of the thermal comfort of broiler chickens during transportation [17,18]. This could be used as a criterion for choosing and improving common practices during pre-slaughter handling, such as the critical time for catching and loading, determination of the density of broilers per crate and wetting of the cargo.

The enthalpy index has been considered an important tool for the characterization of environments and prediction of animal thermal comfort in breeding systems [18,19]. These indexes combine different meteorological variables in their formulas [20], allowing interpretation of the bioclimatic condition of the environment in relation to the animal species [21,22]. For this process, the results are grouped in bands or zones according to pre-established thermophysiological demands given the thermal comfort zone of each species.

Animal welfare during transport is difficult to measure and interpret. Thus, indirect measurements of animal welfare are needed, and one way to proceed with these measurements is using animal-based performance variables such as bruises. Bruises on the carcass are considered an important tool to indicate animal welfare [23], and recording bruises (bruises data) can improve transport conditions and reduce economic losses on future cargo. Carcass bruises can occur during broiler catching or during transport, when the birds are exposed to social changes (such as mixing chickens with different groups from those established during the rearing period) [24,25] and to microclimatic factors that culminate in the crowding of birds in the transport crates [26]. Therefore, transport distance and duration as well as climatic conditions during transport can interfere with broiler welfare and behavior [27] and, ultimately, with their performance [15,28].

Losses from broiler transport are economically significant for the industry. The number of dead broilers recorded on arrival at a slaughterhouse is estimated to be associated with the dynamics of temperature and humidity inside the cargo bay and the duration and distance of transport [29]. A high mortality rate and greater body weight loss have been observed in loads that travelled long distances with long transport periods [30–32]. However, studies have been carried out mainly in temperate countries, with few references showing the thermal profiles of loads in a tropical climate [17] or correlating potential losses with the location of the broilers within the truck trailer.

Studies aiming to elucidate the dynamics of bioclimatic variables within broiler shipments are necessary. The degree of compromise of the thermal comfort of broilers due to environmental conditions and factors such as transport distance and duration is a crucial point in the explanation of yield losses at slaughter. Thus, the objective of this study was to evaluate the microclimate of commercial shipments of broiler chickens transported over different distances during the rainy and dry seasons and the effect of microclimate on slaughter weight, body weight loss, mortality rate and occurrence of bruises on broiler carcasses considering the crate position.

We hypothesized that longer distances, longer transport duration and longer lairage duration can affect broiler chicken performance at slaughter, resulting greater body weight losses. We also hypothesized that broiler chicken transportation, during the summer, can result greater mortality rate and greater bruises on carcass.

## Materials and methods

### Ethical statement

The procedures used in this research were approved by the Ethics Committee on Animal Use of the University of Brasília (University of Brasília; UnB Document Number 130.177/2015).

### Experimental period, animals, management, and transport conditions

The experiment was conducted in the Federal District–Brazil, 15.7939° South, 47.8828° West (Geographic Coordinate System, Latitude/Longitude, Datum WGS84) at an average altitude of 1,130 m and in an A_w_ high-altitude tropical climate according to the Köppen-Geiger climate classification (tropical wet and dry), which has dry winters and hot and humid summers. The average annual temperature is 22°C, and the relative humidity ranges from 20 to 75%. The data collection period covered both the dry (from July to September) and rainy (from November to January) seasons.

Cobb^®^ broilers from Bonasa Alimentos S/A, with a 2.895 ± 0.20 kg average live weight at 48 days of age at slaughter and consisting of male (4 shipments), female (4 shipments), or mixed (4 shipments) broilers, were reared in properly housing systems. Transport details are shown in Table 1. The thermal environment of all sheds was controlled by a ventilation tunnel system. The average density was 12 broiler chickens/m^2^. The broilers had *ad libitum* access to water and a corn and soybean meal-based balanced mash diet. The lighting program was 24 hours on the first day and 23 hours from the second day until slaughter.

**Table 1:** Transport distance, transport duration, truck speed and sex of broilers for each combination of season (Rainy and Dry) and distance (Dist90 and Dist15).

Season	Distance classification	Distance (km)	Duration (h:min)	Truck speed^1^ (km/h)	Sex^2^
Rainy	Dist90	84	02:40	31.50	Male
Rainy	Dist90	72	02:03	35.12	Mixed
Rainy	Dist90	95	02:41	35.40	Female
Rainy	Dist15	15	00:44	20.45	Female
Rainy	Dist15	12	00:21	34.29	Male
Rainy	Dist15	14	00:45	18.67	Mixed
Dry	Dist90	68	01:32	44.35	Male
Dry	Dist90	61	01:42	35.88	Female
Dry	Dist90	158	03:00	52.67	Mixed
Dry	Dist15	18	01:03	17.14	Male
Dry	Dist15	17	00:44	23.18	Female
Dry	Dist15	17	00:36	28.33	Mixed

^1^Average of truck speed

^2^Sex of broilers: male, female and mixed (male and female reared in the same flock).

All shipments occurred in the morning, and detailed information about the shipment conditions (loading duration at the farm (LOADD), transport distance, transport duration (TRANSD), fasting duration at the farm (FFARM), lairage duration (LAIRD), and total fasting duration (FTOTAL) is presented in Table 2.

**Table 2:** Experimental conditions during the shipments. Data are shown as the mean ± sd.

Variable	Shipment	Season					
		Rainy		Dry			
		Distance		Distance			
		Dist15	Dist90	Dist15		Dist90
Temp (°C)	1	27.2	25.0	20.5		21.2
	2	25.1	25.9	16.8		16.4
	3	22.0	19.1	17.0		19.6
	Mean ± SD	24.7 ± 2.1	23.3 ± 3.0	18.5 ± 2.0		19.0 ± 2.8
RH (%)	1	61.0	57.0	49.0		53.0
	2	58.0	60.0	35.0		45.4
	3	82.0	84.0	50.0		32.0
	Mean ± SD	67.0 ± 13.6	67.0 ± 14.9	45.0 ± 8.0		43.5 ± 10.6
LOADD (h:min)	1	32	33	31		42
	2	30	31	35		45
	3	31	35	39		48
	Mean ± SD	31 ± 1	33 ± 2	35 ± 4		45 ± 3
Distance (km)	1	11	80	17		129
	2	15	114	20		71
	3	13	80	18		95
	Mean ± SD	13.0 ± 2.17	91.3 ± 19.6	18.3 ± 1.25		98.3 ± 26.78
TRANSD^1^ (h:min)	1	00:23	01:57	00:54		02:50
	2	00:51	01:46	00:33		01:32
	3	00:37	02:58	00:57		01:52
	Mean ± SD	0:37 ± 0:13	2:14 ± 0:38	0:48 ± 0:14		2:04 ± 0:40
FFARM (h:min)	1	09:11	09:05	08:35		07:00
	2	08:58	08:13	08:22		07:57
	3	07:59	07:28	09:32		08:45
	Mean ± SD	8:42 ± 0:36	8:15 ± 0:48	8:49 ± 0:37		7:54 ± 0:53
LAIRD (h:min)	1	00:41	01:01	02:30		01:22
	2	00:40	01:46	01:32		01:17
	3	01:36	00:20	01:20		02:06
	Mean ± SD	0:59 ± 0:33	1:02 ± 0:44	1:47 ± 0:38		1:35 ± 0:27
FTOTAL^2^ (h:min)	1	10:18	12:44	12:16		11:37
	2	11:24	11:20	11:16		12:31
	3	10:45	12:13	12:24		12:45
	Mean ± SD	10:49 ± 0:35	12:04 ± 0:42	11:59 ± 0:37	12:18 ± 0:35	

TEMP = Environmental Dry Bulb Temperature; RH = Environmental Relative Humidity; LOADD = Loading Duration at Broiler Farm, TRANSD = Transport Duration, FFARM = Fasting Duration at the Farm, LAIRD = Lairage Duration at the Slaughterhouse and FTOTAL = Total Fasting Duration for each Combination of Season (Rainy and Dry) and Distance (Dist15 and Dist90) Classification.

^1^ From the farm to the slaughterhouse

^2^ Adapted from [22].

### Experimental procedures

Overall, 12 shipments were monitored from catching to slaughter during the daytime. The shipments were classified as Dist15 (15 km on average) or Dist90 (90 km on average) considering the routes from the farm to the slaughterhouse. For this, previous study on the geolocation of 54 broiler farms was used, and two clusters with the average distances consistent with those in this study were used.

The broilers were caught by a trained team according to a Japanese method (carried with two hands to hold the wings against the body). The birds were healthy (normal behavior) and dry (after manual inspection) and were sampled arbitrarily. They were transported in crates (Ref. LN77572822, GRANJTEC®, Monte Santo de Minas, MG, Brazil) measuring 73.5 x 53.0 x 21.0 cm (length x width x height). The lairage area was acclimatized and had fans and foggers set at a temperature of 23.5 ± 1.5°C and a humidity level of 84.6 ± 5.3%.

Dry bulb temperature (°C), relative humidity (%), and barometric pressure (mmHg) data during the trial were collected by an automatic weather station in Brasília–DF, Brazil, belonging to the National Institute of Meteorology [33]. These data represent the general environmental conditions on the day of transport and confirm the experimental seasons as dry or wet. It is noteworthy that these data are not the temperature and humidity collected by dataloggers inside the crates, as explained below.

### Assessment of shipments

For the shipments, the duration of ration withdrawal, which corresponded to the beginning of fasting, was recorded. This record allowed the calculation of the FFARM. After catching and placing the broilers in the transport crate, the time spent for complete loading (LOADD) of the crates onto the trucks, the start time (exit from farm) and the time at the end of transport (arrival at the slaughterhouse) were recorded to obtain the TRANSD. The odometer was read prior to departure from the farm and again after arrival at the slaughterhouse to obtain the distance travelled.

At the slaughterhouse, the times when the trucks parked in the lairage area and when they went to the unloading platform were recorded. From these schedules, the LAIRD was obtained, in minutes, for each truck studied. After parking the cargo at the unloading platform, the time of the beginning of broiler slaughter was recorded, which allowed the determination of the FTOTAL for each lot transported. Data are shown in Table 2.

### Transport microclimate assessment and enthalpy comfort index

**Fig 1:**
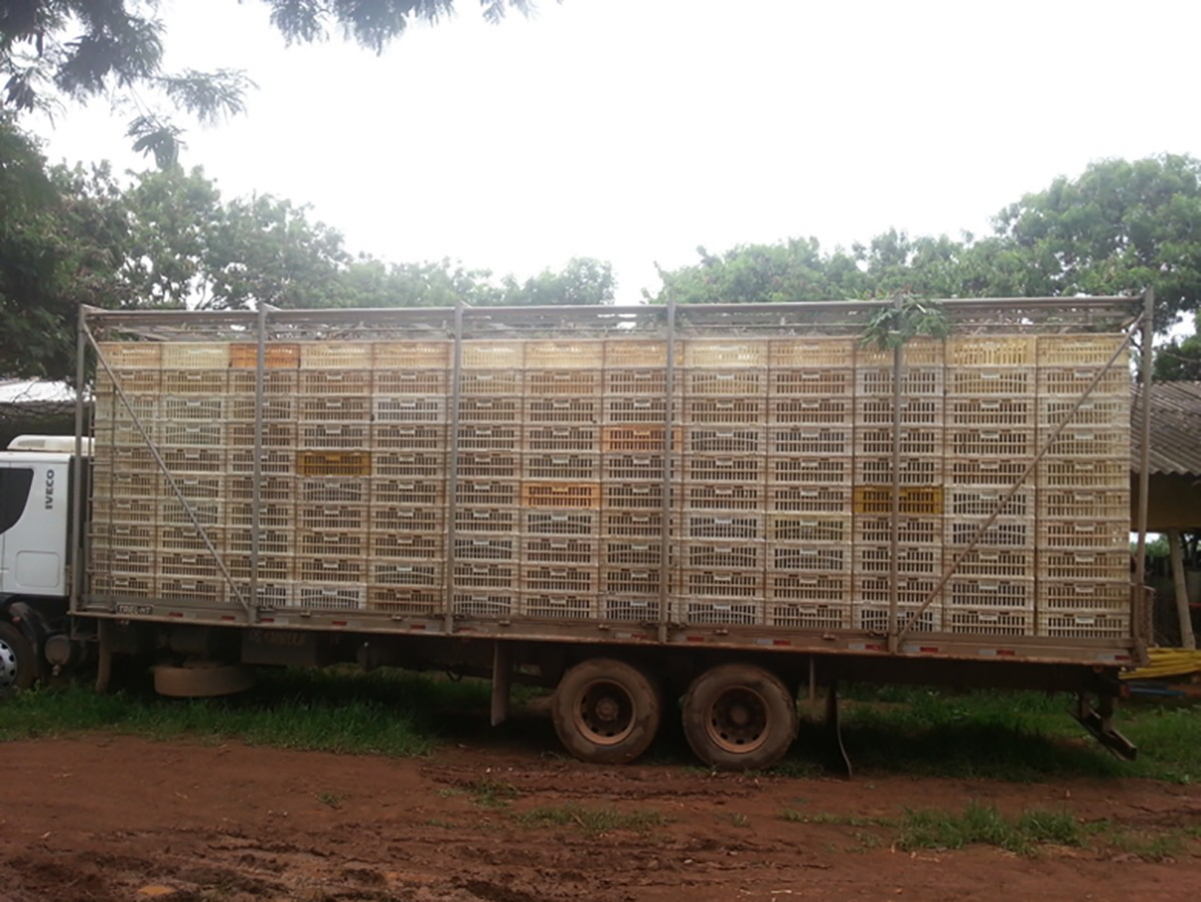
Cargo bay standard used to transport broilers in this study.

Each truck body had 4 rows of crates, 13 crates horizontally and 10 crates vertically, totaling 520 crates (3,640 birds per load) (Fig 1). Twenty-four crates per shipment were fitted with dataloggers (AK170, Akso^®^, São Leopoldo, RS, Brazil), which were fixed inside the crate, locked to avoid unexpected openings, and positioned approximately at broiler height. These crates are called “target crates”. Temperature (T_crate_) and relative humidity (RH_crate_) were monitored for each target crate every 5 minutes during transport.

**Fig 2:**
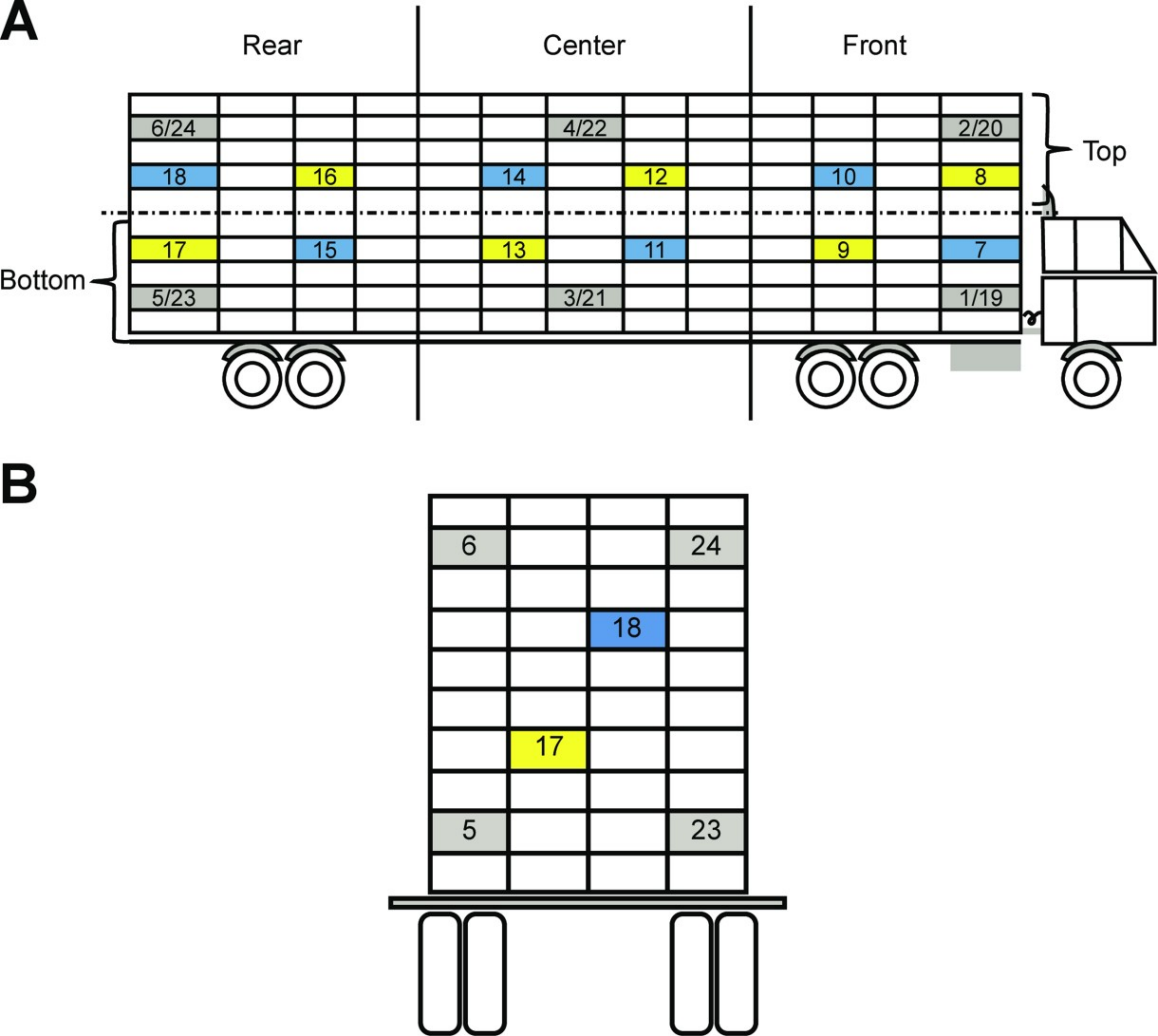
(A) Arrangement of the 24 dataloggers. (B) Rear view, highlighting the center rows. Legend of colors: gray: right and left ends; blue: center right row; and yellow: center left row (adapted from [17]).

The dataloggers were equally distributed such that each section of the truck body (i.e., front, center, and rear) had 8 dataloggers and each half (i.e., top and bottom) had 12 dataloggers (Fig 2). The T_crate_ and RH_crate_ data collected from the 24 dataloggers were used to calculate the enthalpy comfort index (ECI) for each crate. Rodrigues et al. [34] reformulated the ECI equation to consider barometric pressure as stated below. The average barometric pressure during the study was 890 mmHg.

$$h=1.006t+\frac{RH}{p_{b}}10.^{\left(7.5t/237.3+t\right)}.(71.28+0.052t)$$

where:

*h* = Enthalpy index (kJ/kg of dry air);

t = Crate temperature (T_crate_ in °C);

RH = Relative humidity (RH_crate_ in %);

p_b_ = Local barometric pressure (mmHg).

The ECIs were categorized into comfort (35.0 to 48.0 kJ/kg), warning (48.1 to 57.6 kJ/kg), critical (57.7 to 66.1 kJ/kg), and lethal (66.2 to 90.6 kJ/kg) zones for broilers beginning at the sixth week of age based on Queiroz et al. [35].

### Production parameters

To evaluate the initial weight (W_i_), 24 transport crates were weighed, using a calibrated scale (Prix 3 Plus, Toledo^®^, São Bernardo do Campo, SP, Brazil), after catching (7 birds per crate). The loads were not wetted before transport. The weight of each empty crate (tare), measured before transport, was subtracted. To measure the final weight (W_f_), the same crates were weighed after arrival at the slaughterhouse before being unloaded. The difference between the W_i_ and W_f_ (DifW) was calculated in kg/bird. The same calibrated scale (± 0.05 g) was used for all weighing steps and procedures.

The mortality rate (%) or death on arrival (DOA) was obtained by dividing the number of dead birds per crate on arrival by seven (initial number of birds per crate) and then multiplying the quotient by 100. The number of dead birds was recorded after each unloading of the 24 crates in this study. The total mortality in each shipment was also recorded with the support of the slaughterhouse team.

The total mortality (%) for each load was calculated as the ratio of the number of dead birds to the total number of birds transported for slaughter, multiplied by 100. Mortality can be considered the animal's response to (or the consequence of) risk factors for both pretransport management (e.g., chicken fitness or catching method) and conditions during transport, such as TRANSD and distance, climatic conditions, LAIRD at the slaughterhouse and FTOTAL [36]. In this study, pretransport factors were standardized, and the investigation of DOA was used as the key indicator of broiler welfare during transport.

Bruises were evaluated according to the Technical Regulation of the Technological and Hygiene-Sanitary Inspection of Poultry Meat [37] after plucking and evisceration. Arbitrarily, two birds per target crate (for a total of 48 birds per load) were identified (inside the barn, just after catching and before placing the broilers in the transport crate) via plastic bands attached above the tarsal-metatarsal joint. Each bird was identified as having the same number used in the external identification of the crate. This methodology allowed us to evaluate the effect of bird position within the load even after slaughter. The bands were resistant to scalding temperature and the mechanical action of the plunger.

The 48 carcasses were analyzed for bruising on the wings, wing tips, chest and thighs. The prevalence (% with and without bruises), was calculated as described in the Technical Regulation of the Technological and Hygiene-Sanitary Inspection of Poultry Meat [37]. Each bruise was evaluated to assess the relationship between the frequency and crate position in the cargo bay.

### Experimental design

The experiment followed a factorial design [2 seasons (rainy and dry) × 2 distances (Dist15 and Dist90)]. This factorial was then combined with a randomized complete block arrangement, where the ‘blocks’ were the 3 sexes (all males, all females, or mixed shipments). For these 12 combinations (2 seasons * 2 distances * 3 sexes), there was one shipment per combination. For each shipment, 24 crates were recorded at various defined positions (referring to combinations between load sections, parts, and regions); the total of 12 combinations of position factors are shown in Table 3. Thus, the position of the crate can be considered as a split-plot arrangement applied to the shipment.

**Table 3:** Combination of position factors and crate numbers for different load segments, parts and regions.

Positions	Crate numbers	Segments	Parts	Regions
P1	1–19	Front	Bottom	Lateral
P2	2–20	Front	Top	Lateral
P3	3–21	Center	Bottom	Lateral
P4	4–22	Center	Top	Lateral
P5	5–23	Rear	Bottom	Lateral
P6	6–24	Rear	Top	Lateral
P7	7–9	Front	Bottom	Internal
P8	8–10	Front	Top	Internal
P9	11–13	Center	Bottom	Internal
P10	12–14	Center	Top	Internal
P11	15–17	Rear	Bottom	Internal
P12	16–18	Rear	Top	Internal

Therefore, the basic statistical model was a 2*2 factorial (2 seasons * 2 distances), with the effect of sex (all males, all females, or mixed) as another cross-classified fixed effect (‘block’) and the random effect of shipment nested within season, distance and sex. The effect of crate position was considered as a split-plot effect; each shipment was a ‘plot’ and was split into the 12 crate positions.

### Statistical analysis

All statistical analyses were performed using SAS^®^ (v.9.4, Cary, North Carolina). The collected data were subjected to analyses of variance using the PROC MIXED procedure at a 95% confidence level with subsequent comparisons of means using Tukey’s test (5% significance). The dependent variables used were T_crate_, RH_crate_, ECI, W_i_, W_f_ and DifW. In this model, shipment is a random effect, and all the others are fixed. Because shipment is nested within season, distance and sex, it is the ‘error’ term for the corresponding F-tests. The statistical model used is presented below:

$$Y_{ijkm}=\mu +{Season}_{i}+{Distance}_{j}+{Season\times Distance}_{ij}+{Sex}_{k}+{Shipment}_{ijk}+{Position}_{m}+{Season\times Position}_{im}+{Distance\times Position}_{jm}+e_{ijkm}$$

Correlation among quantitative variables was calculated using PROC CORR and then plotted in a heatmap using R software [38,39]. In addition, an analysis of principal components was done in order to identify (graphically) the relationship between data obtained in this study.

The mortality rate and prevalence of bruises on the carcasses were evaluated by the chi-square test using the presence or absence of deaths/lesions as variables, as well as the season and transport distance from the farm to the slaughterhouse as combinations of factors.

## Results and discussion

The shipments travelled, on average, 90 km (Dist90) and 15 km (Dist15). The route taken by the cargoes travelling 90 km was six times longer than that taken by the cargoes travelling 15 km and required three times as long to reach the slaughterhouse. Thus, the TRANSD average was approximately 129 and 43 minutes for Dist90 and Dist15, respectively. The performance of the trucks during transport was directly related to road conditions regarding access to the farms and traffic in urban areas. Thus, the average speed recorded for Dist90 and Dist15 was approximately 40 km/h and 24 km/h, respectively (Table 1). The noise, vibration and air flow in the cargo bay can be influenced by truck speed [40]. However, although we could not measure these variables, they probably play a role in animal welfare, and thus, not all conclusions shown here should be attributed to distance, season, sex or position themselves.

The FFARM of 8:25 hours was excessive according to the Ministry of Agriculture, Livestock and Supply [37], which established an FTOTAL between 6 and 8 hours. The purpose of feed fasting is to promote adequate emptying of the gastrointestinal tract to reduce contamination at the slaughter line during industrial processing [41,42] and thus is required as a sanitary standard. FTOTAL did not substantially exceed the 12-hour limit [43]. When the fasting exceeds 12 hours, FTOTAL can cause intestinal rupture due to intestinal mucosal wear [42]. Thus, reductions in FFARM may result in increased broiler welfare during subsequent stages and a better final slaughter yield [44]. In contrast, FTOTAL less than 8 hours does not allow adequate emptying of the gastrointestinal tract [45], and crop rupture can occur due to the presence of excess food [46]. Therefore, when planning shipments, FTOTAL must be considered a priority and must be taken into account to accomplish the inferior and superior limits.

The average LAIRD was approximately 1:20 hours (Table 2), which was considered appropriate and within the range (1 to 2 hours) stipulated by the Department of Environment, Food and Rural Affairs of the United Kingdom [47]. This range has been described as sufficient to calm broilers and promote a gradual return to homeostasis. The LAIRDs were different because controlling the flow of trucks parked in the lairage area was impossible.

The highest environmental temperature and humidity averages were observed during the rainy season (25.2°C and 67%), while during the dry period, these averages were 19°C and 44.2%, respectively (Table 2). The shed temperature and relative humidity averages were close to the temperature (21 to 23°C) and relative humidity (60 to 70%) ranges recommended by the Cobb broiler management guide [48] for chickens over six weeks old, which was possible because environmental control equipment inside the sheds was turned on during the collection and subsequent placement of broilers in the transport crates.

### Analysis of microclimatic variables

#### Temperature (T_crate_)

Distance and season were not significant factors affecting T_crate_. However, the temperatures registered inside the crates (mean 29.1 ± 2.74°C) were higher than the preconized temperatures for broilers at 48 days of age. Thus, broilers were subjected to heat stress conditions (when animal is outside of thermal neutral zone and, therefore, it needs to spend energy to control its body temperature) for all combinations of season and distance. Mitchell and Kettlewell [49] recommended that the desirable temperature inside the load be below 24°C. Furthermore, Furlan and Macari [8] recommended 23°C.

Theoretically, at long distances, the heat produced by the broilers tends to accumulate inside the load, increasing the internal temperature. Similarly, during short journeys, an increase in internal temperature occurs because the travel duration could be too short to not allow adequate return to homeostasis and thus may produce greater stress in the broilers [50]. Thus, the broilers do not recover from the thermal stress caused by catching and loading, and they maintain an elevated body temperature during transportation, promoting an increase in microclimate temperature [51]. Therefore, at least in part, the T_crate_ registered during the trial could have origin by these mechanisms.

In the rainy season, the average temperature inside the load (T_crate_) was 29.4 ± 3.0°C, and the ambient temperature was 24.5 ± 2.7°C, while in the dry season, these temperatures were 28.8 ± 2.4°C and 18.7 ± 2.4°C, respectively. Thus, thermal differences (between inside and outside of load) of 4.9°C and approximately 10°C were observed in the rainy and dry seasons, respectively. In the United Kingdom, Mitchell and Kettlewell [7] also observed variations between these two environments, with approximately 2 to 5°C of difference for shipments of broiler chickens transported during the summer.

Importantly, even with a thermal difference between the external and internal environments, the temperatures inside the load remained above the thermoneutral zone (which is a range of temperatures at which an animal does not have to actively regulate body temperature) for broilers, regardless of the distance or duration. However, the greater difference between the environments, evidenced in the dry season, could be deleterious for broiler thermal comfort. During this season, stress may have been higher because of thermal amplitude: broilers left a controlled environment inside the barns with a temperature close to 23.0°C, were exposed to an ambient temperature of 19.0°C and later returned to high thermal conditions inside the load (approximately 28.8°C).

**Fig 3:**
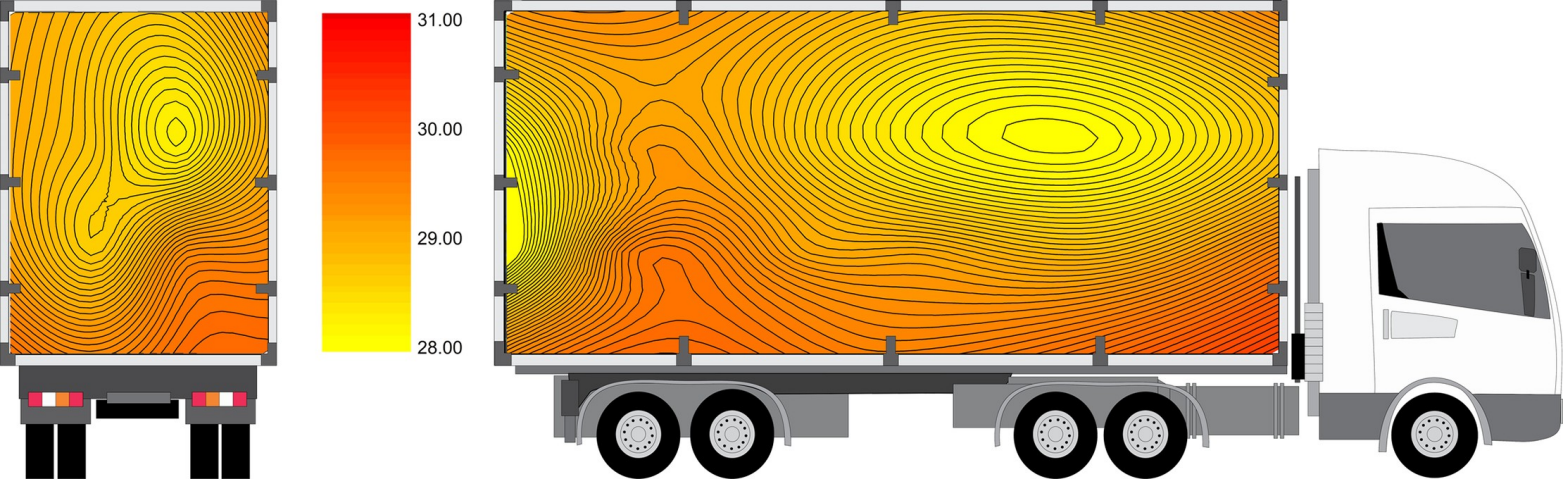
Mean temperature inside the crates (T_crate_) in rear-to-front and lateral views.). The truck is only for illustrative purposes.

T_crate_ was affected (P <0.0001) by crate position (Fig 3). Higher temperatures were observed in the lower levels in the cargo bay. In contrast, lower temperatures were observed in the front and upper parts of the cargo.

**Fig 4:**
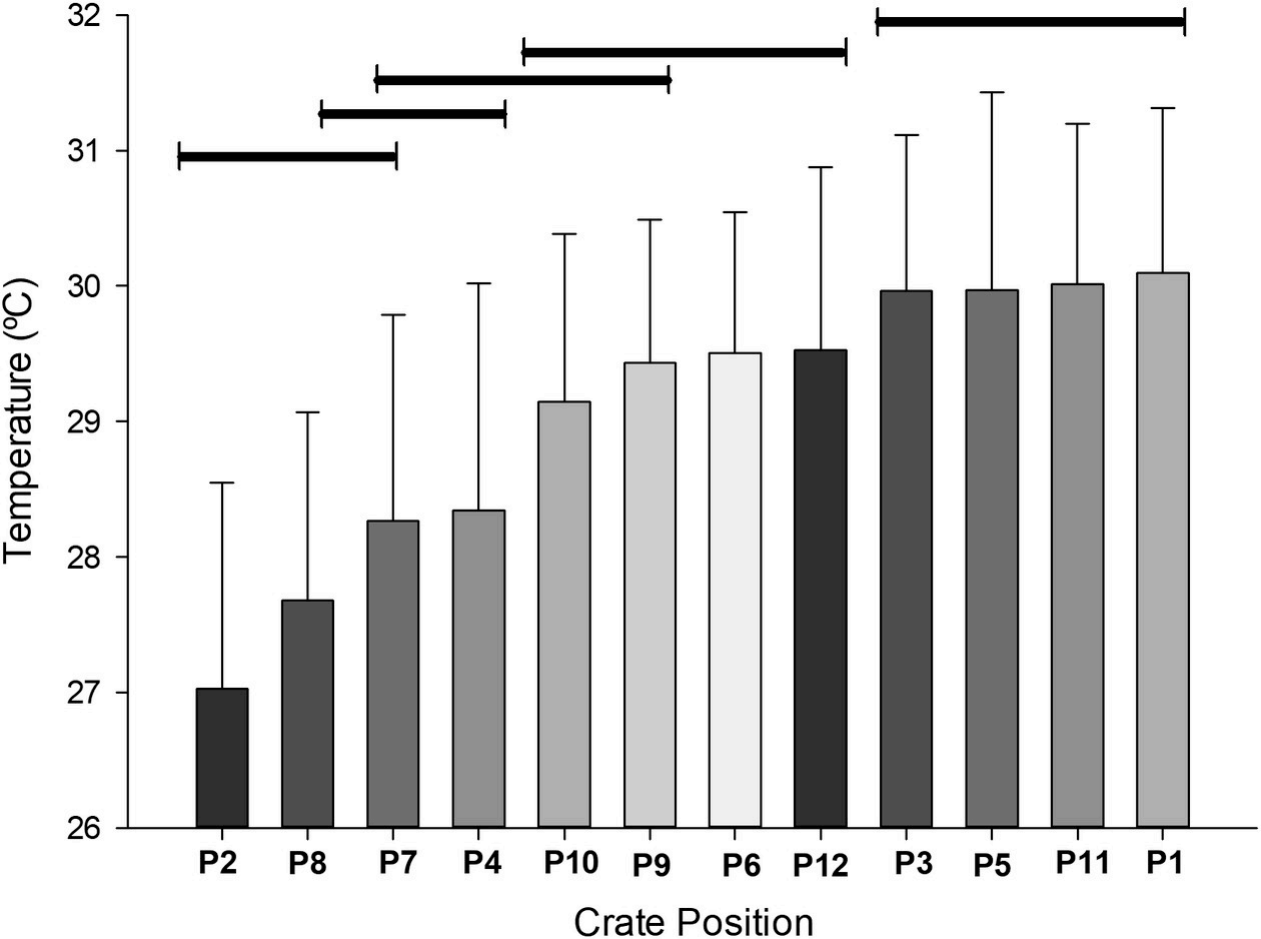
Mean temperature inside the crates according to position. Bars under the same line (top) are not significantly different by Tukey’s test (P<0.05).

A gradual increase in temperature was recorded in a diagonal way (in a lateral perspective), from lower to upper segments and from the front section of cargo (P2, P8 and P7), passing through the center (P4, P10, P9 and P3), and progressing towards the rear of the truck (P6, P12). Thus, P1, P3, P5 and P11 showed higher T_crate_ means (Figs 3 and 4). This heat pattern distribution could be mainly due to an effect caused by truck cabin (impairing the heat dispersion by air movement) or could be due to engine heat production and dispersion. However, futher studies are needed to clarify this.

The results observed here are quite different from the results of Langer et al. [12] and Spurio et al. [16]. According to Langer et al. [12], the temperature inside the vehicle varied from 25°C to 33°C, and the highest temperature was observed at the rear. Spurio et al. [16] measured temperatures in the front (28.8°C), middle (29.4°C) and rear (29.3°C) segments, revealing a variation of 0.5°C between the beginning and end of the load. The difference between the number of observations in these two surveys is noteworthy. Both previously referenced studies used dataloggers to record temperature and other variables at six points along the load, whereas in this study, twelve points were used.

#### Relative humidity

**Fig 5:**
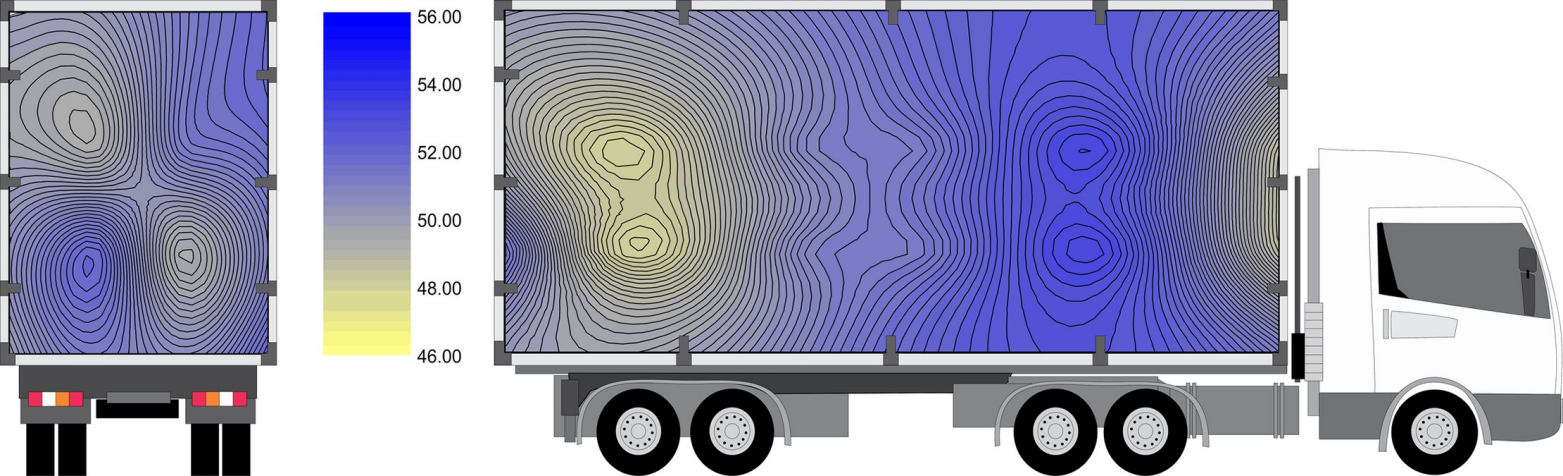
Mean humidity inside the crates (RH_crate_) in rear-to-front and lateral views. The truck is only for illustrative purposes.

**Fig 6:**
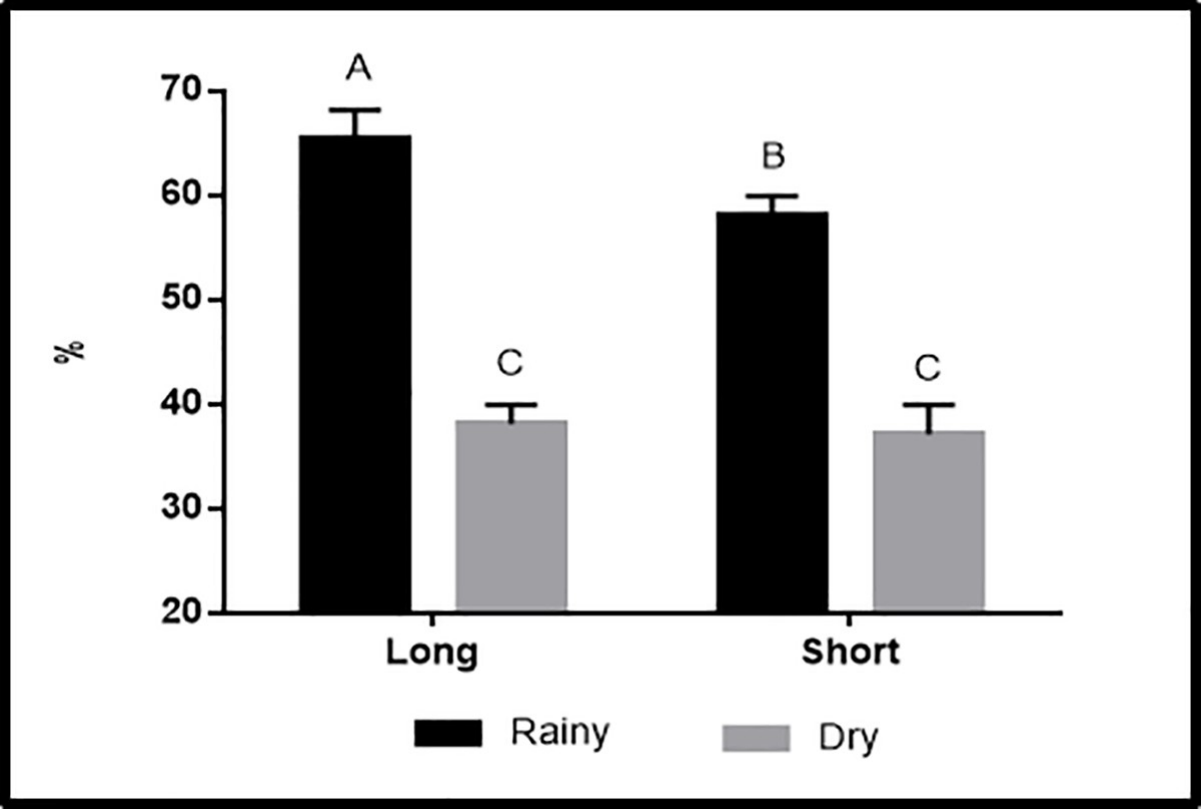
Effect of the interaction between season and distance on load relative humidity (%). ^A^Values with the same capital letters do not differ by Tukey’s test (P > 0.05).

The mean humidity distribution inside the cargo recorded during this trial is shown in Fig 5. There was an interaction (P < 0.0001) between season and distance (Fig 6), with higher relative humidity during the rainy season for Dist90 shipments (65.4 ± 2.7%) than for Dist15 shipments (58.0 ± 1.9%).

Part of the internal humidity comes from the panting of broilers, which tends to increase, especially when they are exposed to the high temperature inside the load, such as that measured during the rainy season for Dist90 shipments (31.1 ± 3.4°C); this moisture accumulates as the broilers travel greater distances to reach the slaughterhouse.

During the dry season, no significant difference was observed in the relative humidity for both distances, with mean values of 38.1 ± 5.8% (Dist90) and 37.2 ± 2.8% (Dist15). These results suggest that for this period, distance itself did not have an effect on the cargo humidity level, which diverged from that observed during the rainy season. This phenomenon could be explained by lower humidity levels during the dry season, which may have allowed the humidity level in the cargo area to move towards the external environment, facilitated by a humidity gradient.

The standard cargo bay used to transport broilers in Brazil is completely open [19] and does not allow the control of microclimatic conditions, making the broilers vulnerable to weather conditions during transport. Modifications to the cargo bay, aimed to increase the air flow inside the load to dissipate the retained moisture, can minimize the thermal discomfort of broiler chickens during transportation [16], and this is more important during the rainy season, as shown here. Further research is needed to develop such vehicles.

#### Comfort enthalpy index

**Fig 7:**
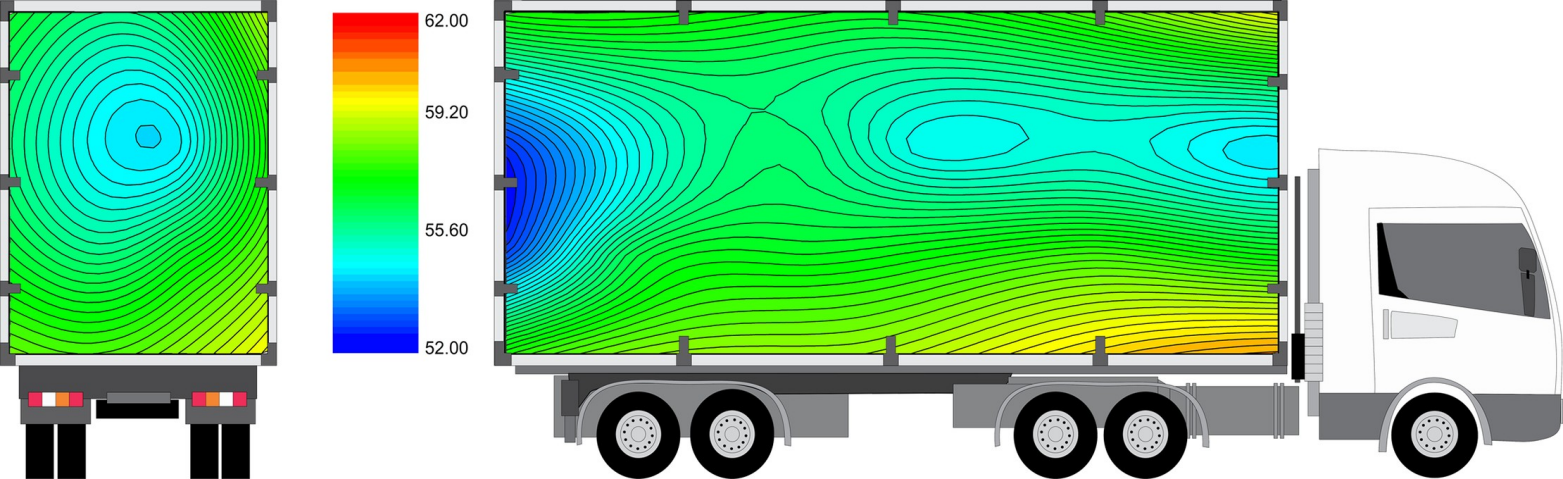
Mean enthalpy comfort index (ECI) in rear-to-front and lateral views. The truck is only for illustrative purposes.

**Fig 8:**
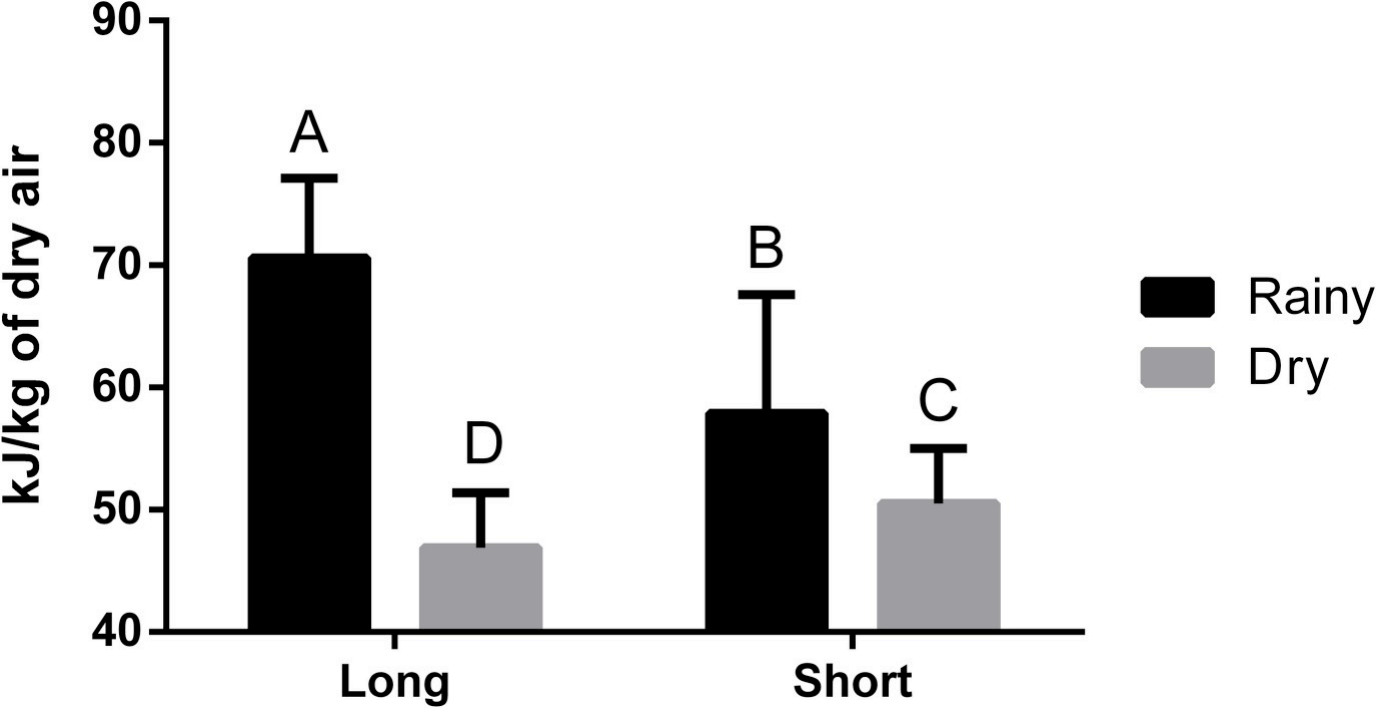
Effect of the interaction between season and distance on load enthalpy comfort index (kj/kg of dry air).

The mean ECI registered during the trial is presented in Fig 7. An effect of the interaction (P < 0.0001; CV = 12.47%) between season and distance on the ECI averages during transport was observed (Fig 8). The ECI indicates the environmental condition in relation to animal heat stress [52], and as the ECI increases, comfort decreases. The highest ECI, which was observed during the rainy season for Dist90 shipments (70.6 ± 6.5 kJ/kg), exceeded the broiler comfort zone and was therefore considered within the lethal zone according to Queiroz et al. [35]. For the same season, the ECI for Dist15 shipments was 58.1 ± 9.7 kJ/kg and thus categorized in the critical zone.

Dist90 during the rainy season, with higher environmental humidity, was harmful to broiler thermal comfort. Filho et al. [18] observed ECI values of 52.0, 72.0 and 65.0 kJ/kg for the transport of broilers during the summer in the morning, afternoon and night, respectively. Afternoon was considered the most critical period for broiler transport, reaching a mortality rate of 0.42%. These findings corroborate the results observed here, as the means observed here often exceed the comfort zone for broilers.

The ECI calculated for Dist90 (46.9 ± 4.5 kJ/kg) during the dry season was within the comfort zone limit. In contrast, the ECI for Dist15 during the dry season (50.5 ± 4.5 kJ/kg) was categorized in the alert zone. Thus, during this season, longer distances may result in improved broiler welfare compared with short distances.

The ECI allows the assessment of compromises in environmental conditions offered to animals during transport for slaughter. However, for better results at the slaughterhouse, other factors, such as the temperature and humidity inside the shed during collection and loading, need to be considered [17]. Moreover, prior knowledge of climatic conditions for the day scheduled for transport may facilitate decision-making concerning the density of broilers per crate as well as wetting the cargo. In addition, an interaction effect between distance and crate position was observed (P = 0.0076). Differences in ECI between positions were observed only on Dist15. However, these differences did not present a specific profile throughout the cargo.

The results observed here for ECI disagree with those presented by Simões et al. [19], who observed a progressive increase in ECI in the load and showed that the center and rear segments were the most problematic for broilers due to the high temperature and humidity conditions observed in the microenvironment. However, these contradicting results could be due to differences in experimental design and procedures. For example, Simões et al. [19] used shorter distances, conducted their experiment in other geographic regions with different climatic conditions and distributed the target crates in a different way in the cargo. Thus, further research is needed to clarify this.

### Analysis of yield variables

#### Age, initial weight, final weight and weight difference

The difference between the ages of broilers in the rainy (48.16 ± 1.86 days) and dry (47.50 ± 1.22 days) seasons was less than one day; therefore, from biological and practical perspectives, this difference was not a significant source of experimental variation. For the distance factor, the mean age was statistically similar (47.83 days). No significant DifW was observed.

**Fig 9:**
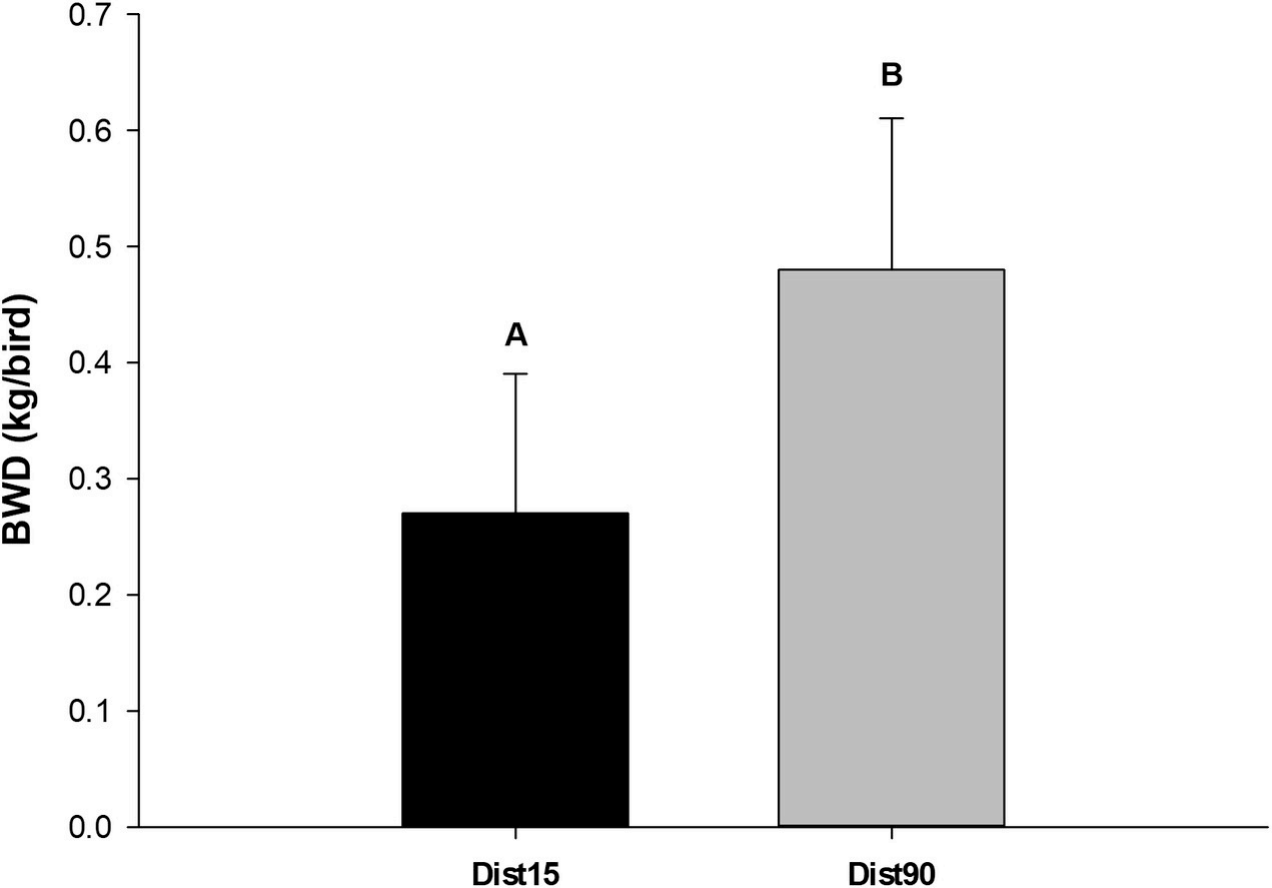
Effect of distance on body weight difference (kg/bird). Different letters indicate significant differences by Tukey’s test (P<0.05).

There was a difference (P = 0.0048) in body weight difference (BWD) between distances (Fig 9). The DifW for Dist15 was lower than that for Dist90, showing that longer distances could be worse for the broilers. For Dist15, BWD was 1.36 ± 0.57% of the W_i_, while for Dist90, it was 2.39 ± 0.63%. Similar results were described by Sowińska et al. [53], who observed values of 1.41%, 2.65% and 2.36% for distances of 100, 200 and 300 km, respectively.

**Fig 10:**
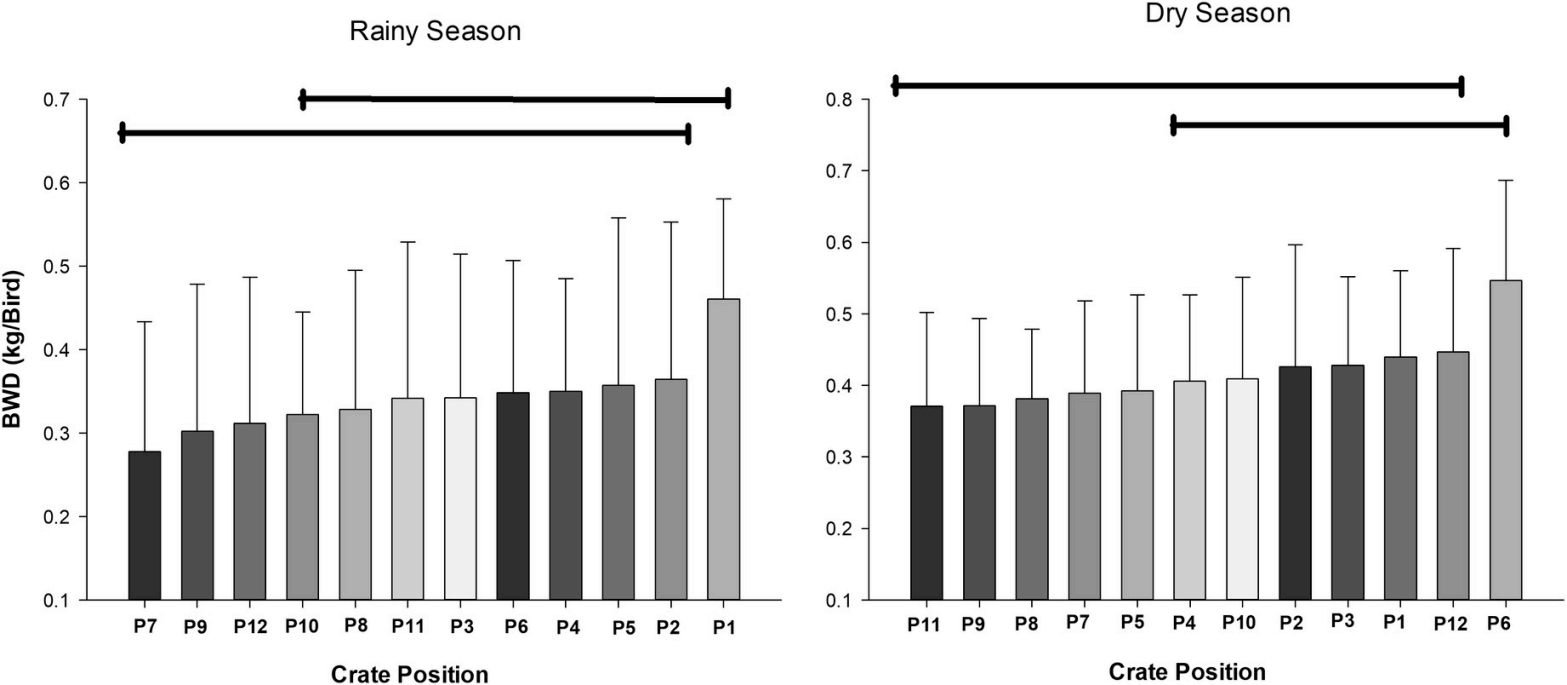
Effect of crate position on broiler chicken body weight difference (kg/bird) according to season. Bars under the same line (top) are not significantly different by Tukey’s test (P<0.05).

An interaction effect between season and position was observed (P = 0.0384). However, these differences did not present specific profiles throughout the cargo for either season (Fig 10).

During the rainy season, the largest BWD was registered for P1, while during the dry season, it was registered for P6. Crates P1 and P6 were diagonally opposite in the cargo bay, and both were located in the lateral region of the load. Curiously, the positions presented differences between the largest BWD values during the rainy (P7, P9, P12) and dry (P11, P9, P8, P7) seasons were in the internal region of the load. In addition, the broilers in the load internal region lost less (in absolute terms, not significantly) body weight (0.049 ± 0.015 kg/broiler) than those in the lateral region (0.060 ± 0.017 kg/broiler). After transportation, the broilers on the side of the truck trailer (P1, P2, P3, P4, P5 and P6) lost 0.011 kg/broiler more body weight. These results reinforce the suggestion that the lateral region may be more vulnerable to air flow speed dynamics. The air flow on the broilers not only allows the W_f_ to decrease but also can cause discomfort (unpleasant situation) and a consequent reduction in welfare during transport, due to the combination with other factors such as: motion, acceleration, vibration, impact and noise [11].

The air flow reaching the upper front of the truck trailer is strong but loses strength towards the rear of the truck [19]. The air movement is able to promote considerable changes within the load, producing thermal gradients that may result in damage to the performance of the broilers [31]. However, this dynamic best represents the air flow in the inner region of the load. In the lateral region, the air flow can be more consistent, and its velocity, although contributing to the dissipation of heat through the removal of internal humidity, can also be a considerable source of stress, resulting in reductions in the yield parameters of these broilers.

BWD had a heterogeneous distribution throughout the load, and this distribution was not significantly correlated with the mean ECI measured during transport at 12 positions along the load. In this study, the positions with a higher ECI in the load did not determine a reduction in performance. Furthermore, position did not reflect a greater loss of body weight of the broilers after transportation. Similar results were observed by Dos Santos et al. [22], who reported that transport distance and season had a greater influence on broiler chicken meat quality than the broiler position in the load.

#### Mortality rate

Mortality during the rainy season was not significantly different between Dist90 (0.19 ± 0.07%) and Dist15 (0.15 ± 0.05%), as shown in Table 4. However, although no significantly difference was seen, the absolute number of dead broilers observed for this period (23 for Dist90 and 17 for Dist15) must receive special attention: from welfare and economic perspectives, these deaths may be important considering the total number of broilers transported during the rainy season in a year.

**Table 4:** Distribution of mortality rate (%) recorded on arrival at the slaughterhouse and tested by a chi-square test.

Distances	Mortality rate (%)	
	Seasons	
	Rainy	Dry
Dist90	0.19	0.22
Dist15	0.15	0.11
P	0.463	0.05

During the dry season, mortality was 0.22 ± 0.04% for Dist90 and lower (P = 0.05) for Dist15 (0.11 ± 0.05%). Shipments of broilers that travelled for 90 km presented a mortality rate twice as high as broilers that travelled for 15 km. This result represented an increase of nine dead broilers over Dist90 journey. Relative humidity during the dry season combined with long transport periods may result in stress conditions inside the truck trailer, resulting in a higher death rate.

However, total mortality recorded during the rainy (0.17%) and dry (0.16%) seasons can be considered satisfactory for both periods. Olivo and Shimokomaki [54] recommended 0.20% as the acceptable limit of posttransport mortality in regions with a tropical climate. Grandin [55] determined an acceptable mortality rate of 0.5% and an excellent value of less than 0.25%. Conversely, special attention should be given to transport that will cover longer distances, especially in the dry season, during which the mortality rate (0.22%) in this study exceeded the recommendations of Olivo and Shimokomaki [54] and approached the limit described by Grandin [55].

In studies carried out in Brazil, Vieira et al. [14] observed a mortality rate of 0.12% for distances of 25 to 50 km and of 0.41% for those over 51 km, and Silva et al. [56] registered values of 0.16% and 0.27% for loads during the summer without wetting that travelled distances of 15 and 55 km, respectively. Aral et al. [57] analyzed 846 broiler loads in Turkey and observed mortality exceeding the recommended limits for all treatments evaluated, with an average of 0.29% for the group from 0–120 minutes, 0.38% from 121 to 240 minutes and 0.40% from 241 to 360 minutes. These data confirmed that the longer the transport distance and duration were, the lower the viability of broilers recorded upon arrival at the slaughterhouse [15,58].

In this trial, mortality rates were not affected by crate position. Thus, the difference in ECI caused by crate position (Fig 7) was not sufficient to cause death. However, according to Mitchell et al. [59] and Kettlewell and Mitchell [60], regions of high temperature and humidity in the cargo may reflect a higher prevalence of death, as seen by Filho et al. [17], who observed higher mortality rates in the center and bottom part of the truck trailer, where high temperature and humidity were measured.

#### Bruising of carcasses

The prevalence of wing bruising was not influenced by season and distance factors (Table 5). The total prevalence observed was 5.38% for the rainy season and 5.00% for the dry season. These results are within the limit reported by Grandin [61], who categorized "normal" variations from 5 to 6% of the total number of broilers slaughtered. However, the author noted that this percentage decreased as better broiler management practices were used, reaching values of less than 1% [62]. Costa et al. [63] reported greater damage caused by wing bruises in broiler carcasses transported longer distances, which were 43.67% for an average distance of 250 km.

**Table 5:** Percentage of distribution of wing, wing tip, breast and thigh bruises among season (rainy and dry) and distance (dist90 and dist15) analyzed by a chi-square test.

Cuts	Rainy		Total	P	Dry		Total	P
	Dist90	Dist15			Dist90	Dist15		
Wing	6.25	4.81	5.38	0.475	4.81	5.19	5.00	0.475
Wing tip	10.58	17.95	15.00	0.021	11.54	9.09	10.32	0.316
Breast	0.96	4.49	3.08	0.106	2.56	3.90	3.23	0.506
Thigh	7.21	10.90	9.42	0.158	2.88	3.06	3.06	0.793

A significant difference (P < 0.021) in red wing tip prevalence was observed between the short and long distances in the rainy season. The broilers transported the short distance presented a prevalence of red wing tip of 17.95%, which was approximately 7% greater than that of broilers transported the long distance (10.58%). The prevalence of red wing tip is usually associated with both the catching method and hanging of the broilers in the slaughter line [64] but the transport cannot be ruled out as one of the possible causes. The flapping of the wings at the moment of hanging at the entrance of the desensitization vat increases the blood flow to the end of the wings, which is retained even after exsanguination.

A red wing tip devalues a carcass and is less acceptable to consumers [64]. Considering these results, better conditions in the broiler collection environment (interior of the shed) and reception/hanging area (slaughterhouse) are recommended. It’s known that catching (on shed) and reception (on slaughterhouse) are critical points causing red wing tip. Thus, better conditions in the broiler collection environment and the reception area at slaughter are always recommended. Low levels of lighting in these environments soothe broilers, reduce wing flutter and ease handling. Likewise, the use of blue light promotes greater comfort for broilers and workers who handle broilers with greater care and, thus, reduce damage to carcasses [65]. In addition, maintaining air conditioning with the use of fogging fans is recommended for improving the thermal sensation of broilers in both environments [14].

However, considering the results observed here, there is something more during rainy season causing red wing tip in Dist15 journeys. Perhaps, during rainy Dist15 shipments there are not enough time to soothe broilers and leave them find a suitable and comfortable position inside the crate can make broilers more active and reactive in the reception at slaughterhouse, causing red wing tip due broiler struggles. In the same way, very short journeys can allow broilers arriving in the slaughterhouse rested (or not tired) and thus, more reactive, allowing them to flapping the wings more intensely. In contrast, in Dist90 shipments, broilers could find a good position to travel and arrives in the slaughterhouse more soothe, causing less lesions or, the journey in Dist90 shipments could be longer enough to tire the broilers, which struggle less and with less energy, reducing their injuries. The distance, TRANSD and LAIRD (Table 2) corroborate with these as rainy Dist15 shipments travelled shorter distances (13.0 ± 2.17 km), with shorter transport duration (37 ± 13 min) and presented shorter LAIRD (less then 1 hour), however, this should also receive further study.

No significant effect of season and distance on bruises on the breast and thighs was observed. The mean breast bruising prevalence was 3.08% for the rainy season and 3.23% for the dry season, while the thigh bruising prevalence was 9.42% and 3.06%, respectively. The maximum percentage of recommended thigh bruising is 1%, with 0.5% being considered excellent [55]. This bruising is also associated with catching management on farms. When broilers are caught by one or both legs, this score tends to increase. Placing the broilers upside down in the transport crate favors an increase in this type of bruising and may lead to limb fractures in some cases.

The prevalence of bruising on carcasses is mainly associated with the management of the broilers during the breeding period. These injuries occur usually during the final period of breeding, when the broilers are heavier. About 30 to 50% of bruises occur during collection, and 20 to 35% occur post-collection [66]. Moreover, determining the impact of transport on the percentage of carcass lesions is quite difficult. Thus, the collection of the broilers in sheds must be constantly monitored to correct any problems. Due to the repetitive nature of this activity, employees often catch the broilers by their leg(s), increasing injuries to the broilers.

#### Correlation analysis and principal components analysis

Correlation analysis (Table 6) showed a relationship between position and T_crate,_ corroborating the results presented in the analysis of microclimatic variables section. In addition, position was also related to BWD, which was described and discussed above. BWD also showed a positive relationship with transport distance, TRANSD and LAIRD, contributing to the thesis of longer distances, (and consequently) longer TRANSD and longer LAIRD with greater body weight losses.

**Table 6:** Correlation between the following variables: crate position, crate temperature (T_crate_), crate relative humidity (RH_crate_), crate enthalpy comfort index (ECI_crate_), initial weight (W_i_) of broilers, final weight (W_f_) of broilers, body weight difference (BWD) of broilers, loading duration (LOADD) at the farm, transport distance (DIST) from the farm to the slaughterhouse, transport duration (TRANSD) from the farm to the slaughterhouse, lairage duration (LAIRD) at the slaughterhouse, fasting duration at the farm (FFARM), and total fasting duration (FTOTAL).

Variables	Position	T_crate_	RH_crate_	ECI_crate_	W_i_	W_f_	BWD	LOADD	Dist	TRANSD	LAIRD	FFARM
T_crate_	0.158^*^											
RH_crate_	0.014 ns	-0.188^*^										
ECI_crate_	0.124 ns	0.559^**^	0.707^*^									
Wi	0.002 ns	-0.699 ns	-0.198^*^	-0.269^**^								
Wf	0.015 ns	-0.074 ns	-0.193^*^	-0.263^**^	0.994^**^							
BWD	-0.120^*^	0.026 ns	-0.056 ns	-0.072 ns	0.255^**^	0.151 ns						
LOADD	0	-0.296^**^	-0.205^*^	-0.441^**^	0.282^**^	0.267^**^	0.197^*^					
Dist	0	-0.167^*^	-0.045 ns	-0.123 ns	0.108 ns	0.061 ns	0.450^**^	0.556^**^				
TRANSD	0	0.067 ns	-0.042 ns	0.069 ns	0.018 ns	-0.027 ns	0.414^**^	0.379^**^	0.946^**^			
LAIRD	0	-0.096	-0.263^**^	-0.426^**^	0.288^**^	0.249^**^	0.414^**^	0.590^**^	0.096 ns	-0.042 ns		
FFARM	0	0.341^*^	-0.485^**^	-0.154^*^	-0.180^*^	-0.146 ns	-0.348^**^	-0.359^**^	-0.635^**^	-0.533^**^	-0.185^*^	
FTOTAL	0	0.237^*^	-0.773^**^	-0.436^**^	0.097 ns	0.067 ns	0.296^**^	0.417^**^	0.090^**^	0.094 ns	0.669^**^	0.411^**^

^*^ P < 0.05

^**^ P < 0.0001; ns = No significance

**Fig 11:**
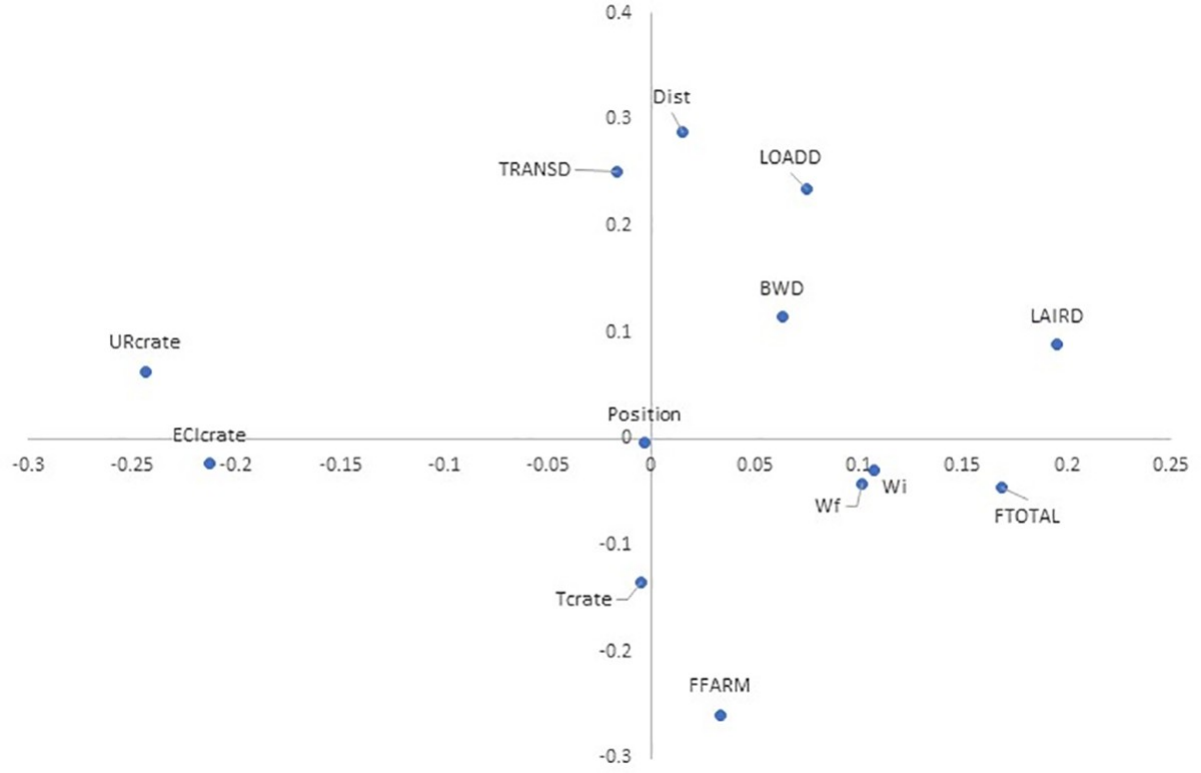
Principal component analysis of parameters measured during the shipments. Crate position (Position), crate temperature (T_crate_), crate relative humidity (RH_crate_), crate enthalpy comfort index (ECI_crate_), initial weight (W_i_) of broilers, final weight (W_f_) of broilers, body weight difference (BWD) of broilers, loading duration (LOADD), transport distance (Dist), transport duration (TRANSD), lairage duration (LAIRD), fasting duration (FFARM), and total fasting duration (FTOTAL).

The first eigenvector explained 27.13% of the data, whereas the second eigenvector explained 22.14%. Principal component analysis (Fig 11) showed a high correlation between RH_crate_ and ECI_crate_ as well as transport distance and TRANSD.

## Conclusions

In terms of comfort, the rainy season was the most critical period for broiler transport, resulting in the highest ECI. For example, in the rainy season and Dist90, the highest ECI was ranked in the lethal zone. Broiler chickens presented a higher body weight difference (BWD) when transported over longer distances but crate positions with higher ECI in the load did not reflect significant body weight loss and mortality. Thus, load microclimate can compromise broiler chicken welfare without necessarily impair broiler productivity. The prevalence of bruising on carcasses was not affected by the interaction between season and distance. Possibly, this prevalence can be associated with the management of the broilers during the breeding period. Broiler chicken performance, during transport, can be also related to road conditions, being hard to evaluate the actual impact of seasons and distances on animal welfare.
